# Human regulatory proteins associate with non-coding RNAs from the EBV IR1 region

**DOI:** 10.1186/s13104-018-3250-8

**Published:** 2018-02-20

**Authors:** V. S. Tompkins, D. P. Valverde, W. N. Moss

**Affiliations:** 1 Roy J. Carver Department of Biochemistry, Biophysics, and Molecular Biology, Iowa State University, 2437 Pammel Drive, Ames, IA 50011 USA; 2 Department of Molecular Biophysics and Biochemistry, Yale University School of Medicine, New Haven, CT 06536 USA

**Keywords:** IR1, EBV, ncRNA, lncRNA, sisRNA, EBNA-LP, HNRNP, NONO, FUS, HuR, LIN28

## Abstract

**Objective:**

The function of Epstein–Barr virus (EBV) stable intronic sequence (sis)RNAs, non-coding RNAs transcribed from a region required for EBV-mediated cellular transformation, remain unknown. To better understand the function of ebv-sisRNA-1 and ebv-sisRNA-2 from the internal repeat (IR)1 region of EBV, we used a combination of bioinformatics and biochemistry to identify associated RNA binding proteins. The findings reported here are part of ongoing studies to determine the functions of non-coding RNAs from the IR1 region of EBV.

**Results:**

Human regulatory proteins HNRNPA1 (heterogeneous nuclear ribonucleoprotein A1), HNRNPC, HNRNPL, HuR (human antigen R), and protein LIN28A (lin-28 homolog A) were predicted to bind ebv-sisRNA-1 and/or ebv-sisRNA-2; FUS (fused in sarcoma) was predicted to associate with ebv-sisRNA-2. Protein interactions were validated using a combination of RNA immunoprecipitation and biotin pulldown assays. Both sisRNAs also precipitated with HNRNPD and NONO (non-POU domain-containing octamer-binding protein). Interestingly, each of these interacting proteins also precipitated non-spliced non-coding RNA sequences transcribed from the IR1 region. Our findings suggest interesting roles for sisRNAs (through their interactions with regulatory proteins) and provide further evidence for the existence of non-spliced stable non-coding RNAs.

**Electronic supplementary material:**

The online version of this article (10.1186/s13104-018-3250-8) contains supplementary material, which is available to authorized users.

## Introduction

Stable intronic sequences (sis)RNAs and long non-coding (lnc)RNAs are non-coding RNAs (nc)RNAs known to perform many functions that can regulate gene expression [1, 2]. Aberrant expression of lncRNAs is thought to contribute to disease states such as cancer [3]. The impact of the relatively new category of sisRNAs are not well understood but could include roles in development or cancer [1, 4]. Rarely (if ever), is the function of these RNAs independent of the proteins that bind them. Numerous RNA binding proteins have been identified and many of their important functions have been discussed elsewhere in detail [5], including their implication in disease states such as cancer [6].

Two abundant sisRNAs expressed from the internal repeat (IR)1 region, containing the W-repeats, of the Epstein–Barr virus (EBV; human herpes virus 4, HHV4) genome were identified previously [7]. Their transcription is linked to that of the Epstein–Barr nuclear antigen leader protein (EBNA-LP), and occurs during cancer-associated latency III infection as well as in a rare latency program found in Burkitts lymphomas: Wp-restricted [8]. Efficient transformation of B cells requires EBNA-LP expression [9]. Indeed, the connection between EBV and cancer is well established [10, 11], but the role of the IR1 region stable introns in cellular transformation is not. Recently, a mutation in ebv-sisRNA-1 that inhibited EBV-mediated transformation of B cells was reported [12]. Though the mutation did not occur in isolation, this maintains the idea that the ncRNA from this region is important for tumorigenesis. Here, we extend the understanding of the non-coding RNAs, ebv-sisRNA-1 and ebv-sisRNA-2, through the demonstration of their association with a number of human regulatory proteins. Further, we provide evidence that these intronic sequences are more likely to be found embedded within non-spliced transcripts that retain both exons of the IR1-region.

## Main text

### Methods

Descriptions of cell lines and transductions, biotin precipitations and mass spectrometry, bioinformatics and data processing, RNA immunoprecipitation, RNA extraction, and PCR are in Additional file 1.

### Results and discussion

#### Initial identification and validation of protein interactors of ebv-sisRNA-1

**Fig. 1:**
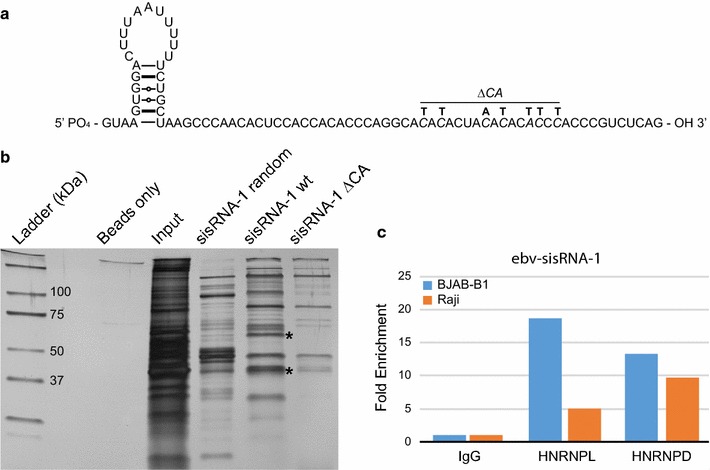
Interaction of ebv-sisRNA-1 with HNRNPL and HNRNPD. **a** Schematic illustration of the structure of ebv-sisRNA-1. Base pairing is depicted by lines and circles. The mutations present in the ΔCA form are indicated. **b** Silver stained gel after biotinylated precipitation from whole BJAB lysate. The ebv-sisRNA-1 wild-type (sisRNA-1wt) form was compared to either a randomized sequence, which represents background non-specific RNA binding proteins, or the mutated ΔCA form. Bands excised and subjected to mass spectrometry are denoted with asterisks. **c** Fold enrichment of ebv-sisRNA-1 following RIP using antibodies against HNRNPL, HNRNPD, or normal rabbit IgG from either BJAB-B1 or Raji cells. Data represent the mean of two independent RIPs per cell line (one for BJAB-B1 HNRNPD) and are normalized to control IgG. See Additional file 3 for individual RIP data point values

The majority of ebv-sisRNA-1 is predicted to be unstructured (Fig. 1a), providing easier access to single-stranded RNA binding proteins. To identify proteins that bind ebv-sisRNA-1, biotin precipitation assays were performed using BJAB cell lysates (EBV negative) and either a wild-type or deletion mutant of the conserved CA-rich region (ΔCA). This region was speculated previously to be important for heterogeneous nuclear ribonucleoprotein (HNRNP)L binding [7]. Silver staining revealed losses of several protein bands, prominently at both 60–65 kDa (size of HNRNPL) and 35–40 kDa (most intense band for wt) compared to ΔCA mutation (Fig. 1b). Mass spectrometry of these regions identified HNRNPL and HNRNPD/AUF1 as the most probable interactors (Additional file 2). HNRNPL binding is fully consistent with reported CA-rich consensus binding sequences [13, 14]. HNRNPD/AUF1 typically binds AU-rich elements (AREs) of RNA [15] but has also been shown to interact with HNRNPL [16]. RNA immunoprecipitation (RIP) for HNRNPL and HNRNPD/AUF1 on both Raji and BJAB-B1 cells, which harbor type 1 and type 2 EBV genomes, respectively, revealed an association with ebv-sisRNA-1 (Fig. 1c; Additional file 3). Ebv-sisRNA-1 is highly abundant and expressed at the same time as EBNA-LP [7], and recent genetic evidence suggests that EBNA-LP and possibly ebv-sisRNA-1 may be important for EBV-mediated B cell transformation [12]; thus, its interactions with two key regulatory proteins have interesting implications.

HNRNPD/AUF1 is best known for its function in regulating mRNA abundance (e.g., binding to ARE-containing 3′ untranslated regions (UTRs) and destabilizing transcripts) [17]. Overexpression of HNRNPD/AUF1 can promote oncogenesis and control translation [18, 19]. Interestingly, HNRNPD/AUF1 binds directly to EBER1 [20], another EBV ncRNA that is highly expressed throughout all stages of latent/lytic infection. The proposed function of this EBER1 interaction is to perturb the normal homeostasis between HNRNPD/AUF1 and target mRNAs in EBV-infected cells. Given that both ebv-sisRNA-1 and EBER1 are abundantly present during the initial patterning of B-cell gene expression (e.g., latency III), it is enticing to speculate that ebv-sisRNA-1 and EBER1 may coordinately contribute to disease-promoting functions of HNRNPD/AUF1.

HNRNPL has a proposed role in various cancers and is known to affect the expression (e.g., alternative splicing) of a wide array of human genes [21–24]. HNRNPL shuttles between the cytoplasm and nucleus; thus, its interaction with a viral ncRNA that is abundantly present in the nucleus, suggests a potential role in disrupting HNRNPL localization/interactions. This could have a vast impact on the alternative splicing of many host genes; as well as, interestingly, in the regulation of the EBNA-LP gene from which ebv-sisRNA-1 is derived: e.g., through a competition between EBNA-LP pre-mRNA and the accumulating sisRNA that forms stable ribonucleoproteins (RNPs) with HNRNPL. These possibilities are being explored.

#### Identification of additional interactors of ebv-sisRNA-1 and -2

**Fig. 2:**
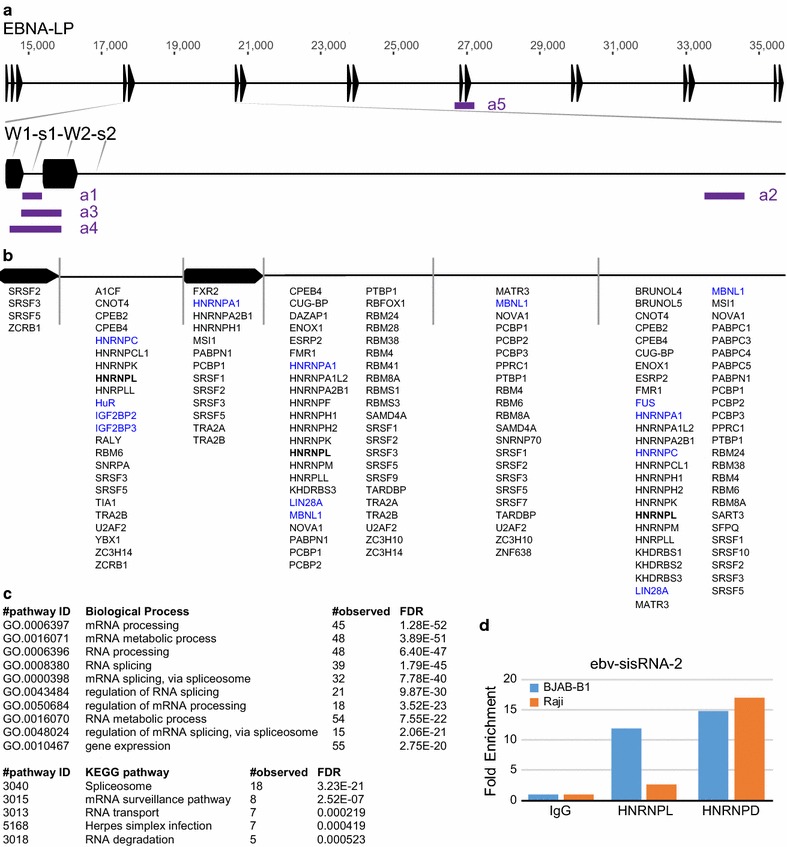
RNA protein binding profile from one W-repeat of the IR1 region. **a** Schematic illustration of the EBNA-LP locus (top) with internal W-repeats. The region used for RBPmap [25] is enhanced (bottom, at scale) and shows the W1 and W2 exons compared to the sisRNA (s1 or s2) introns. Regions amplified for PCR (*a*mplicon) in these studies are shown in purple: a1—ebv-sisRNA-1, a2—ebv-sisRNA-2, a3—sisRNA-1 to W2, a4—W1 to W2, and a5—across both exons. **b** RNA binding proteins resulting from RBPmap are indicated by region (above, not to scale). Blue indicates proteins assayed by RIP in addition to HNRNPL (bold). **c** Top results from STRING [38] analysis for enrichment in biological process (top) and KEGG pathway; false discovery rate (FDR) is given. **d** Fold enrichment of ebv-sisRNA-2 after RIP using HNRNPL, HNRNPD, or normal rabbit IgG antibodies and either BJAB-B1 or Raji cells. Data represent the mean of two independent RIPs per cell line (one for BJAB-B1 HNRNPD) and are normalized to control IgG. See Additional file 3 for individual RIP data point values

To better understand the protein binding repertoire of ebv-sisRNA-1 and ebv-sisRNA-2, we used the program RBPmap [25] to predict human host RNA binding proteins (RBPs) that might directly interact. Both type 1 (NC_007605) and type 2 (NC_009334) EBV sequences were analyzed using a sequence fragment of the IR1 W-repeat region from each genome, which contains exon W1, ebv-sisRNA-1, exon W2, and ebv-sisRNA-2 (Fig. 2a, W1s1W2s2). RBPmap compares these sequences to known consensus binding sites of human and mouse proteins and classifies/ranks hits based on their fit to the consensus motif. 81 unique proteins were identified that had significant binding site consensus throughout the region of interest (Fig. 2b; see Additional file 4 for binding region detailed information). The putative interacting proteins are known to be involved in biological processes of RNA processing, splicing, and gene expression (Fig. 2c; Additional files 3 and 5).

Consistent with our findings above, ebv-sisRNA-1 contained predicted HNRNPL binding regions (Fig. 2b). Ebv-sisRNA-2 did as well, and this interaction was verified by RIP (Fig. 2d). Similar to ebv-sisRNA-1, ebv-sisRNA-2 precipitated with HNRNPD/AUF1. Multiple interaction sites for several RBPs throughout the sisRNAs suggest similar functions for potential regulation of EBNA-LP and sisRNA processing, as well as downstream effects on cellular homeostasis of RBP interactions with endogenously regulated host genes, which remains to be explored.

#### Validation of select interactors

**Fig. 3:**
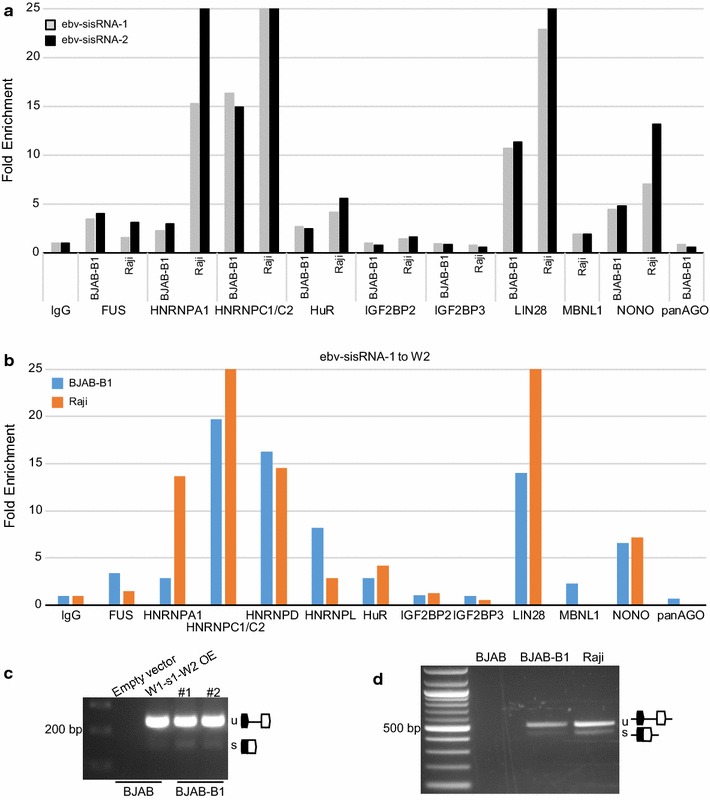
RIP binding of ncRNA from EBV W-repeat region. **a** Fold enrichment for ebv-sisRNA-1 or ebv-sisRNA-2 after RIP using antibodies against the indicated proteins from BJAB-B1 or Raji cells. **b** Fold enrichment for ebv-sisRNA-1 to exon W2 (a3 from Fig. 2a) after RIP using antibodies against the indicated proteins from BJAB-B1 or Raji cells. For all RIPs, data represent the mean of mostly two independent experiments per cell line (where only one experiment was done is shown in Additional file 3) and data are normalized to control IgG. For both A and B, data are cut off at a fold enrichment value of 25. **c** RT-PCR (a4 from Fig. 2a) from either lentiviral transduced BJAB using empty vector or a W1 through W2 overexpression (OE) construct or from two independent RNA isolations from BJAB-B1 cells. The amplified forms of the unspliced (u) or spliced (s) products are indicated. **d** RT-PCR (a5 from Fig. 2a) from BJAB, BJAB-B1, or Raji total RNA across the exon/intron boundaries (a5 from Fig. 2a) for retention of sisRNA-1. The amplified forms of the unspliced (u) or spliced (s) products are indicated

Eight other predicted proteins (Fig. 2b, blue; Additional file 6) were tested for their ability to co-precipitate either ebv-sisRNA-1 (HuR/ELAVL1, IGF2BP2, IGF2BP3), ebv-sisRNA-2 (FUS, HNRNPA1, LIN28, MBNL1), or both (HNRNPC). Quantitative (q)PCR for both sisRNAs was performed for all RIPs (Fig. 3a; Additional file 3). HuR, HNRNPA1, LIN28, and HNRNPC precipitated both ebv-sisRNAs with at least twofold enrichment over normal IgG in two cell lines. FUS bound ebv-sisRNA-2 in both cell lines, but only precipitated ebv-sisRNA-1 in BJAB-B1 cells. IGF2BP2, IGF2BP3, and MBNL1 did not bind under these experimental conditions.

Two RBPs not predicted by RBPmap were also tested. To determine if sisRNAs were potential targets of micro (mi)RNAs (e.g., acting as “sponges”), an antibody that targets multiple Argonaute proteins was used. Neither sisRNA was enriched in our experiments. This result challenges a previous in silico predicted interaction [26] of ebv-sisRNA-1 and miR-142-3p (previously proposed to be targeted by another herpes virus ncRNA [26, 27]). Lytic EBV ncRNAs originating from the OriP region contain long hairpin stems similar in size/structure to ebv-sisRNA-2 [7, 26] and were found to interact with the paraspeckle-associated protein p54nrb/NONO [28]. The OriP ncRNA was found to promote transcription/replication of EBV and to modulate host innate immunity through its interactions with paraspeckles. Likewise, NONO interacts with EBER2 [29] and may bridge an interaction with host transcription factor PAX5, which supports EBV replication through recruitment by EBER2 to the viral genome [30]. Similar to the OriP and EBER2 ncRNAs, NONO bound both ebv-sisRNAs by RIP. The common interaction of these viral ncRNAs with NONO suggests potential overlapping functions (e.g., replication or immune modulation) that need to be tested. Additionally, localization of the sisRNAs (and other viral ncRNAs) to paraspeckles remains to be shown.

These results show binding—either direct or indirect—of the IR1 sisRNAs to HNRNPL, HNRNPD/AUF1, HuR, HNRNPA1, LIN28, NONO, and HNRNPC (Fig. 3a). Intriguingly, each of these proteins are implicated in pro-cancer-related phenotypes [6, 31]. Binding of these regulatory proteins to ebv-sisRNAs may play roles in the emergence of these phenotypes in EBV-associated diseases, which merits further investigation.

#### Identification of a junction-spanning IR1-derived ncRNA that co-precipitates with RBPs

Intrigued that both ebv-sisRNAs associated with HuR, HNRNPA1, FUS, and LIN28, when only one or the other was predicted to bind, a primer set that spanned the ebv-sisRNA-1 to W2 splice junction was tested (Figs. 2b, amplicon 3; 3b; Additional file 3). This RNA, containing an intact splice junction, precipitated with the majority of interacting proteins, suggesting that these RBPs also bound non-spliced RNA from the W-repeat region. To test for the relative abundance of spliced to non-spliced ebv-sisRNA-1, RT-PCR was performed using a forward primer targeting exon W1 and a reverse primer targeting exon W2 (Figs. 2b, amplicon 4; 3c). The overwhelming majority of RNA contained intact exon–intron junctions. The non-spliced form was also more abundant than the spliced form in EBV-free BJAB cells transduced to overexpress W1-sis1-W2. Likewise, amplification from the 3′ end of sisRNA-2 across W1-sisRNA-1-W2 to the 5′ end of the next sisRNA-2 intron (Fig. 2b, amplicon 5) demonstrated that the predominant form of RNA from the IR1 W-repeats retains intact exon–intron boundaries (Fig. 3d).

There are several possible interpretations of these findings. Firstly, W-repeat intronic RNAs could be embedded within non-polyadenylated [poly(A)] lncRNAs arising from this region. Secondly, splicing may have resulted in excision of an entire stable sis2-exonW1-sis1-exonW2 region (~ 5.9 kb) or an exonW1-sis1-exonW2-sis2 (~ 3 kb) region; the stability of such products could possibly be conferred through circularization and back splicing [32]. Both interpretations are made plausible by early work that found prominent Northern blot bands of poly(A)− RNA up to 13 kb in size. [33]. Thirdly, splicing may be inhibited leading to the accumulation of intron-containing pre-mRNA from the EBV W-repeat region, which has been observed previously [34]. HNRNPL and HNRNPA1 are known spicing repressors [35], which supports this hypothesis. Likewise, lncRNAs spanning the W-repeat intronic regions may be polyadenylated. Previous analyses of poly(A)+ samples [33, 36], showed Northern blot bands overlapping poly(A)− RNAs as well as larger ones (up to ~ 22 kb). These possibilities are not mutually exclusive and the exact architecture of the ncRNAs from this region is likely to be complex: consisting of multiple sized species of spliced/unspliced RNAs with and without poly(A) tails. Supporting this idea, recent analyses of RNA-Seq data from EBV-infected cells found extensive read coverage of the ebv-sisRNA regions in both ribosome-depleted and poly(A)-selected samples [37].

#### Concluding remarks

We have shown that the ebv-sisRNAs exist within a complex milieu of non-coding transcripts. In addition to independent intronic units, sisRNAs are embedded within various sized ncRNAs that can arise from alternative splicing or initiation and that may be polyadenylated. Regardless of their transcript structure, sisRNAs are capable of interacting with a number of human regulatory proteins. We present a list of 81 proteins from in silico predictions and validate six highly interesting ones that associate with both ebv-sisRNA-1 and ebv-sisRNA-2 (FUS, HNRNPA1, HNRNPC, HNRNPL, HuR, LIN28). Furthermore, we identify additional interactions with HNRNPD/AUF1 and NONO, both of which interact with other EBV ncRNAs. Taken together, our results begin to unravel the RNP composition of this intriguing class of viral ncRNAs and suggest multiple leads for follow-up analyses into the functional consequences of deduced interactions: e.g., in EBV infection and the emergence of disease.

## Limitations

Additional work is needed to confirm other predicted interactions as well as to determine if validated binding proteins are interacting directly or indirectly. Most importantly, the exciting implications of the identified/validated RBPs have yet to be explored. We hope this report will facilitate additional research required to understand the functions of these interesting EBV ncRNAs.

## Additional files

**Additional file 1.** Methods and materials. Detailed description of methods and materials used to collect and analyze data.

**Additional file 2.** Mass spectrometry results. List of proteins that bound biotinylated forms of ebv-sisRNA-1 after mass spectrometry from gel slices indicated in Fig. 1B. Hits resulting from either the top (60-65 kDa) or bottom (35-40 kDa) are indicated.

**Additional file 3.** Fold enrichment individual experimental results from all RIPs.

**Additional file 4.** RBPmap of EBV W1s1W2s2. Human or mouse proteins predicted (RBPmap) to bind W1s1W2s2 RNA (see Fig. 2A) from the IR1 region of EBV. Results from both EBV type 1 and type 2 sequences are included as well as a comparison.

**Additional file 5.** STRING analyses of predicted RBPs. Results from STRING enrichment analyses of 81 RBPmap-identified proteins. Information includes annotations and enrichment data on biological processes, KEGG pathways, molecular functions, cellular components, InterPro, and Pfam.

**Additional file 6.** Map of predicted binding sites for proteins assessed by RIP.

## Abbreviations

EBV: Epstein–Barr virus
EBNA-LP: Epstein–Barr virus nuclear antigen leader protein
FDR: false discovery rate
HHV4: human herpesvirus 4
IR1: internal repeat 1
lncRNA: long non-coding RNA
ncRNA: non-coding RNA
poly(A): polyadenylated
qPCR: quantitative polymerase chain reaction
RBP: RNA binding protein
RNP: ribonucleoprotein
RT-PCR: reverse-transcriptase polymerase chain reaction
sisRNA: stable intronic sequence RNA
UTR: untranslated region
