# Supplementary material for: Human regulatory proteins associate with non-coding RNAs from the EBV IR1 region
Source: BMC Res Notes. 2018 Feb 20;11:139. doi: 10.1186/s13104-018-3250-8 (PMC5819218; doi:10.1186/s13104-018-3250-8)
Supplement: Supplementary file 1 — Additional file 1. Methods and materials. Detailed description of methods and materials used to collect and analyze data. [file 13104_2018_3250_MOESM1_ESM.docx]

**Methods and Materials:**

**Human regµlatory proteins associate with non-coding RNAs from the EBV IR1 region**

Tompkins, V.S.^1^, Valverde, D.^2^, and Moss, W.N.^1^*

^1^Roy J. Carver Department of Biochemistry, Biophysics, and Molecµlar Biology, Iowa State University, 2437 Pammel Drive, Ames, IA, 50011, USA.

^2^Department of Molecµlar Biophysics and Biochemistry, Yale University School of Medicine, New Haven, Connecticut 06536, USA.

*Correspondence: [wmoss@iastate.edu](mailto:wmoss@iastate.edu).

**Methods and Materials**

*Cell lines and transduction*

Cells were a kind gift from Joan Steitz (BJAB, BJAB-B1) or Siegfried Janz (Raji). All cells were maintained in a humidified incubator at 37°C, 5% CO_2_. Growth medium was RPMI supplemented with 2mM L-glutamine, 1% penicillin-streptomycin, 10 mM Hepes, 1 mM sodium pyruvate (all from Life Technologies), and 10% FBS (Atlanta Biologicals). Exon W1 to exon W2 was amplified from genomic DNA of BJAB-B1 cells and cloned into pLenti-puro (Addgene) using PstI and XhoI sites (see **Primers Table** below). Expression in BJAB cells was obtained using lentivirus transduction. Lenti-X cells (Clontech Laboratories) in 6-well dishes (VWR) were co-transfected with 12 μg pLenti-puro vector DNA, 6 μg psPAX2, and 3 µg pMD2.G packaging vectors (Addgene) using the CaPO_4_ method. Medium was changed 4-6 h post transfection. Supernatant was harvested 48 h after transfection, filtered using PVDF (0.45 μm; Millepore Sigma). Virus was freshly prepared for each use. 5 × 10^5^ BJAB cells were plated in one-fourth cµlture medium and three-fourths viral supernatant with 7.5 µg/ml polybrene (Sigma Aldrich). Plates were centrifµged (1200×g, 1.5 h, ambient temperature) then allowed to sit 30 min. Cells were pelleted (160×g, 10 min), plated in 3 ml fresh medium, and incubated (37 °C, 5% CO_2_) for two days. Puromycin (2 µg/ml) selection was applied for 2 days followed by maintenance dosing (1 µg/ml) for subsequent cµlture.

*Biotin precipitation and mass spectrometry*

DNA oligonucleotides containing the sisRNA-1 wt, randomized and CA-disrupted (ΔCA) construct sequences, plus a T7 promoter sequence were obtained from IDT. Oligos were heated to 95 °C then slow cooled to RT in a thermocycler. In vitro transcription reactions (25 µl) were set up combining 1 µg of the annealed oligonucleotide templates with 2 µl DTT (100 mM; Life Technologies), 0.5 µl RNase OUT (Life Technologies), 4 µl of NTP mix (25 mM; NEB), 2.5 µl of Biotin-11-CTP (10 mM; Trilink), 2.5 µl 10X T7 buffer (0.5 M Tris-HCl pH 7.5, 100 mM MgCl2, 20 mM spermidine), and 3 µl T7 RNA polymerase. After a 2 h incubation, samples were DNase treated then purified by Phenol/Chloroform extraction and ethanol precipitation.

For each precipitation, 3 µg of RNA was diluted into 45 µl of water and heated to 90 C, then snap cooled on ice. 5 µl 10X RNA structure buffer (100 mM Tris pH 7, 1 M KCl, 100 mM MgCl2) was added and the RNA allowed to incubate at RT for 20 min. The annealed RNA was nutated for 1 hr with 1 ml of nuclear extracts (~10^7^ BJAB cells homogenized via Dounce homogenizer with RIP buffer [150 mM KCl, 25 mM Tris pH 7.4, 0.5 mM DTT, 0.5% NP-40, 1 mM PMSF, plus protease inhibitor {Sigma})]. 50 µl of streptavidin beads (pre-washed with RIP buffer) were added and nutation continued for 1 additional hr. Beads were spun down at 1000 x g (2 min at 4 °C) and washed 5X using 500 ml cold RIP buffer. Beads were then heated to 100 °C in 50 µl SDS gel loading buffer for 5 min then fractionated on a 4–20% precast polyacrylamide gel (Bio Rad). Gels were silver stained using a silver stain kit (Pierce) and bands of interest excised.

Excised bands were sent to The Mass Spectrometry (MS) & Proteomics Resource of the W.M. Keck Foundation Biotechnology Resource Laboratory (Yale University School of Medecine) for destaining and analysis via LC-MS/MS (Tandem mass spectrometry) Protein Identification using an Orbitrap mass spectrometer. Fragment patterns were analyzed vs. SwissProt_2014 via MASCOT database search.

*Bioinformatics – RBPmap and STRING*

EBV type 1 (NM_007605) and type 2 (NM_00934) sequences from a W1 throµgh sisRNA-2 of the IR1 region (1: 17626-20697; 2: 17607-20676) was inputted to RBPmap [1]. Defaµlt parameters with archived human and mouse consensus protein binding sequences were used. Input tables were downloaded and modified for analyses (see Additional file 3). The graph derived from these data (see Additional file 5) was generated using Microsoft Excel.

Single protein lists were generated from RBPmap and inputted to STRING (Snel et al. Huynen, 2000) to determine enrichment of biological processes and pathways. Tables were downloaded and modified for analyses (see Additional File 4).

*RNA immunoprecipitatio*n

Cells were washed once with DPBS (Life Technologies) before lysis (2×10^7^ c/ml) in RIPA buffer (50 mM TrisHCl, pH 8.0, 1 mM EDTA, 150 mM NaCl, 1.0 % NP-40, 1.0 % Sodium Deoxycholate, 0.1 % SDS) containing HALT protease inhibitors (Thermo Fisher, 1 to 100 dilution), 1 mM PMSF, 350 U/ml RNaseOUT (Invitrogen). The suspension was sonicated briefly (5 sec at 50% amplitude) to enhance solubility before centrifµgation (14,000×g for 10 min 4°C) to precipitate insoluble components. The lysate was diluted 2.5-fold with lysis buffer lacking detergent (50 mM TrisHCl, pH 8.0; 1 mM EDTA; 150 mM NaCl, 80 U/ml RNaseOUT, 1mM PMSF) prior to pre-clearing with 100 μl Protein A/G-sepharose per 10^7^ c (SantaCruz sc2003) for 1 hr at 4°C. The following antibodies/antisera were incubated for 2 hr at 4° while mixing: 2 µg each of p54nrb/NONO (sc376865), LIN-28 (sc374460), HNRNP C1/2 (sc32308), IGF2BP2 (sc377014), IGF2BP3 (sc365640), HuR/ELAVL1 (sc5261), HNRNPA1 (sc32301), MBNL1 (sc47740), Normal Mouse IgG (sc2025), or 5 µg FUS (sc47711) from Santa Cruz; 2 µg panAGO (Millipore Sigma MABE56), HNRNPL (Bethyl Laboratories, A303-895A), or normal rabbit IgG (Cell Signaling 2729s); 10 µl HNRNPD/AUF1 (αC; c/o Joan Steitz [2]). Then 25 µl of Protein A/G-PLUS agarose (Santa Cruz sc2003) was added and incubated 1 hr at 4°C while mixing. The beads were washed at least three times with DPBS containing 20 U/ml RNAse OUT and 1 mM PMSF 1 mM (1 ml each wash followed by a 5 sec vortex on high, centrifµgations were 1000×g 3 min at 4°C. Trizol (1 ml; Invitrogen) was then added to the beads and 100 µl of lysate (25% input) for RNA extraction.

*RNA extraction and PCR*

RNA was extracted using the Trizol method according to the manufacturer’s protocol (Invitrogen). In brief, Trizol mixtures (1 ml) were added to phase-lock gel tubes (QuantBio, 2302830) and allowed to sit 5 min at room temperature. 200 µl chloroform was added, the tube was shaken vigorously for 15 sec, and incubated for 2 min at room temperature prior to centrifµgation (12,000×g 10 min 4°C). The top layer was transferred to new tube and 1.5 µl of Glycoblue (Invitrogen, AM9515) and 500 µl isopropanol was added, mixed, and incubated for 10 min at room temperature). RNA was pelleted by centrifµgation (12,000×g 10 min 4°C), washed with 1 ml 70-75% ethanol (vortexed briefly), and pelleted again (7500×g 5 min 4°C). The pellet was briefly dried and suspended in H_2_O. DNA was removed using DNAse I (Thermo Fisher, AM2222; 37°C 30 min), followed by inactivation (0.5 µl EDTA added, 75°C for 10 min), and RNA clean up using the Zymo RNA Clean and Concentrator-5 (R1015) per the manufacturer’s protocol. The input samples were suspended in 2.5 times the amount of H_2_O as the immunoprecipitated ones, and equal volumes were added to reverse-transcriptase (RT) reactions for a 10% total input. RT was performed with Superscript III (Invitrogen) using random hexamer priming. Quantitative PCR (qPCR) was performed using PowerUP Sybr mix and the QuanStudio3 instrument (Thermo Fisher) for 40 cycles. All runs contained no-RT and no template controls and were performed with three technical replicates. Primer sequences can be found in the **Primers** **Table** below. RT-PCR was performed from cells lysed directly in Trizol and isolated as above. Two µg total input was used for these RT reactions prior to PCR with Dream Taq (Thermo Fisher) and visualization using ethidium bromide on a 1.5 or 2% tris-acetate EDTA (TAE) agarose gel. All RT reactions and non-qPCR was done using the SimpliAmp thermocycler (Thermo Fisher). Nucleic acids were quantified using NanoDrop ONE (Thermo Fisher). A Fotodyne (Fotodyne Inc.) imaging station was used to obtain images.

*Data Processing*

Microsoft Excel was used to generate graphs and process the qPCR data. Sequence analyses was done using Geneious version R11 (<https://www.geneious.com>; Biomaters) [3] and IGV_2.3.90 [4]. Figures were created using Adobe Photoshop and Illustrator.

**Primers Table.** Primers used in cloning and PCR. All sequences are 5' PO_4_- to -OH 3'

| **Cloning EBV type I segment W1-sisRNA-1-W2** |  |  |  |
| --- | --- | --- | --- |
|  | FWD | GCAGCCTGCAGGCCTAGGGGAGACCGAAGTG |  |
|  | REV | GCAGCCTCGAGCCCTGAAGGTGAACCGCTTACC |  |
|  |  |  |  |
| **qPCR primers** |  | **Sequence** | **Amplicon** |
| ebv-sisRNA-1 | FWD | GTAAGTGGACTTTAATTTTTTCTGCTAAGCCC | a1 |
|  | REV | TGGGTGTGTGTAGTGTGTGC |  |
| ebv-sisRNA-2 | FWD | CGTTGCTAGGCCACCTTCTCAG | a2 |
|  | REV | CATTTGTGTGGACTCCTGGCG |  |
| sis1 to W2 | FWD | GCAGCCTGCAGGCCTAGGGGAGACCGAAGTG | a3 |
|  | REV | CTGGACGAGGACCCTTCTAC |  |
| **RT-PCR primers** |  |  |  |
| W1 to W2 | FWD | CCGAAGTGAAGTCCCTGGAC | a4 |
|  | REV | CTGGACGAGGACCCTTCTAC |  |
| sis2 3' to sis2 5' | FWD | GGTCTTCTACCTCTCCCTAGCC | a5 |
|  | REV | CCT AAA CCC ACC CAG ACT AGC C |  |

**References**

1. Paz I, Kosti I, Ares JM, Cline M, Mandel-Gutfreund Y. Rbpmap: A web server for mapping binding sites of rna-binding proteins. Nucleic Acids Res. 2014;42:W361-W7.

2. Lee N, Pimienta G, Steitz JA. Auf1/hnrnp d is a novel protein partner of the eber1 noncoding rna of epstein-barr virus. RNA. 2012;18:2073-82.

3. Kearse M, Moir R, Wilson A, Stones-Havas S, Cheung M, Sturrock S, et al.Drummond A. Geneious basic: An integrated and extendable desktop software platform for the organization and analysis of sequence data. Bioinformatics. 2012;28:1647-9.

4. Robinson JT, Thorvaldsdóttir H, Winckler W, Guttman M, Lander ES, Getz G, Mesirov JP. Integrative genomics viewer. Nat Biotechnol. 2011;29:24.
