# Supplementary figures and images for: Human regulatory proteins associate with non-coding RNAs from the EBV IR1 region
Source: BMC Res Notes. 2018 Feb 20;11:139. doi: 10.1186/s13104-018-3250-8 (PMC5819218; doi:10.1186/s13104-018-3250-8)

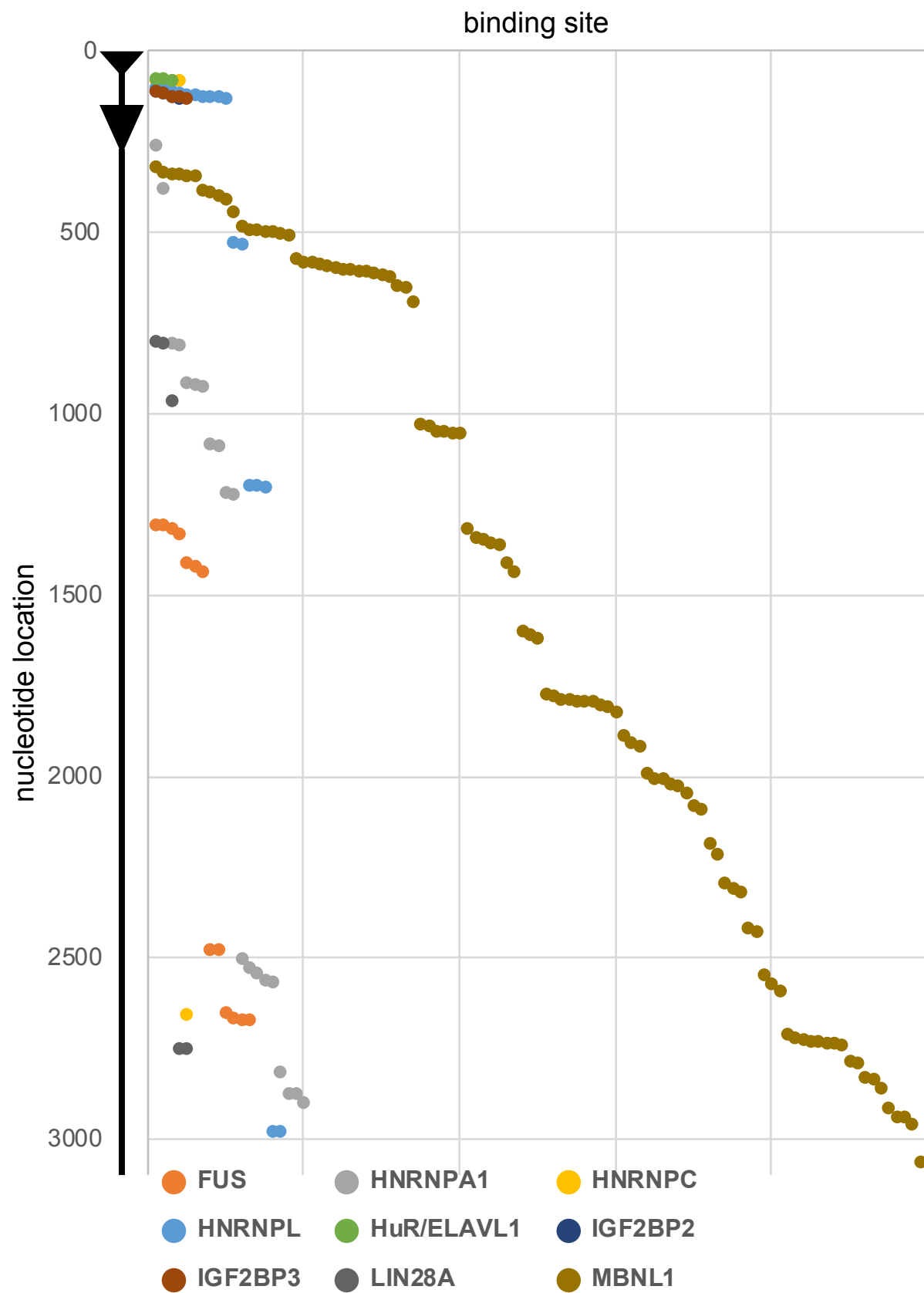

Supplement: Supplementary file 6 — Additional file 6. Map of predicted binding sites for proteins assessed by RIP. [file 13104_2018_3250_MOESM6_ESM.pdf]
